# Editorial: *Bifidobacteria* and Their Role in the Human Gut Microbiota

**DOI:** 10.3389/fmicb.2016.02148

**Published:** 2017-01-06

**Authors:** Francesca Turroni, David Berry, Marco Ventura

**Affiliations:** ^1^Laboratory of Probiogenomics, Department of Life Sciences, University of ParmaParma, Italy; ^2^Division of Microbial Ecology, Department of Microbiology and Ecosystem Science, University of ViennaVienna, Austria

**Keywords:** bifidobacteria, genomics, gut microbiota, probiotic bacteria, metagenomics

Bifidobacteria were originally isolated by Tissier at the beginning of the last century from infant stool samples and until now 57 (sub)species have been included in this bacterial genus (Turroni et al., 2011; Milani et al., 2014). Bifidobacterial biology has captured increasing attention in the last 15 years due to widespread interest in using bifidobacteria as health promoting microorganisms, i.e., known as probiotics, in the food industry. Significant efforts have been expended to dissect the genetics as well as molecular mechanisms underlying the probiotic action(s) of bifidobacteria. This has led to the establishment of a new scientific discipline called probiogenomics, which is providing new insights into the diversity and evolution of bifidobacteria and to the identification of their health-promoting effector molecules (Ventura et al., 2009; Turroni et al., 2014). Furthermore, thanks to recent discoveries about the microbial diversity of the human gut, we have started to achieve detailed insights about the composition of the bifidobacterial communities in this complex ecosystem and to understand the intricate relationship with their host as well as with the other members of the gut microbiota. Altogether, this knowledge will be crucial in order to develop novel bacterial therapeutic strategies based on bifidobacteria.

The 21 articles comprising the Research Topic “Bifidobacteria and their role in the human gut microbiota” illustrate the many key advances that now define our understanding of bifidobacteria-host molecular interactions, as well as the relationship between the various members of the *Bifidobacterium* genus with other residents of intestinal microbiota.

The current knowledge of the general features of bifidobacteria is reviewed, with particular focus on the metabolic features used to colonize the human gastrointestinal tract (O'Callaghan and van Sinderen) and on the specific molecular mechanisms employed by these microorganisms to interact with host tissue (Ruiz et al.; Wei et al.; Westermann et al.). In addition, the compositional changes of bifidobacterial populations associated with different stages of life are reviewed (Arboleya et al.). Variations in the composition of the human gut microbiota and bifidobacterial communities due to subsistence strategy, i.e., from hunter-gatherer to urban industrial Western lifestyle, has been studied (Soverini et al.). Also, the canine gut microbiota and the contribution of bifidobacterial taxa in this ecosystem have been explored (Sabbioni et al.).

There is growing interest in the mechanisms utilized by bifidobacteria to interact with each other in the gut ecosystem. These include specific metabolic foraging features related to glycans used for cross-feeding (Turroni et al.) as well as metabolic strategies used by bifidobacteria to assimilate nitrogen in their natural ecological niche (Ferrario et al.). The current knowledge regarding compounds that may positively influence human gut microbiota composition, such as short-chain fatty acids (SCFAs), is reviewed (Rivière et al.). Notably, these microbial end-products possibly allow the co-existence of bifidobacterial strains with other butyrate-producing bacteria in the human colon.

Another important feature of bifidobacterial physiology is their ability to restrain pathogen growth in the intestine. This feature is investigated by two original research articles specifically focused on the competition with *Escherichia coli* O157:H7 as well as *Salmonella enterica* serovar Typhimurium (Bondue et al.; Vazquez-Gutierrez et al.), and *Clostridium difficile* (Valdés-Varela et al.). Furthermore, an investigation of the anti-viral effect of bifidobacteria toward noroviruses has been included (Li et al.).

In addition, the genomics of the order *Bifidobacteriales* has been explored via a phylogenetic and comparative study on proteins from all publicly available genome sequences belonging to the members of this order (Zhang et al.). Such analyses provide an in-depth overview of their evolutionary relationships and identify molecular markers that are unique to the different members of the order *Bifidobacteriales* at multiple phylogenetic levels. Moreover, Brandt and Barrangou propose a phylogenetic analysis of the *Bifidobacterium* genus using glycolysis enzyme sequences as a typing method.

The role of the gut microbiota in metabolism and metabolic disease risk has been described (Connolly et al.). The ecology of bifidobacteria has been also discussed with an opinion article focusing on the contribution of bifidobacteria to the infant gut microbiota in humans (Tannock et al.). The efficacy of the probiotic *Bifidobacterium animalis* subsp. *lactis* species, which is widely used in fermented dairy products, in the management of gastrointestinal disorders, has been investigated. In particular, two studies have been included, one investigating the effect of this species on intestinal barrier strength (Martín et al.) and another analysing the mitigating role of exopolysaccharides of *B. animalis* subsp. *lactis* in intestinal inflammatory processes such as ulcerative colitis (Hidalgo-Cantabrana et al.). Finally, technological strategies to preserve and protect cell viability of probiotic bifidobacteria that are needed to guarantee the efficacy of probiotic products are amply illustrated in this Research Topic (Yeung et al.).

The integration of various studies to define gut microbiota composition coupled with detailed analyses of the physiology and genomics of bifidobacteria will be crucial in order to improve our understanding of the complex interactions occurring in the human gut. Altogether, these data will undoubtedly help in developing the industrial use of bifidobacteria including for therapeutic use.

## Author contributions

All the authors listed have made substantial, direct and intellectual contribution to this work and approved it for publication.

### Conflict of interest statement

The authors declare that the research was conducted in the absence of any commercial or financial relationships that could be construed as a potential conflict of interest.
